# Valproate-induced hyperammonemic encephalopathy. A case report

**DOI:** 10.1192/j.eurpsy.2022.1860

**Published:** 2022-09-01

**Authors:** A. Cerame, A. Franco Soler, P. Coucheiro, R. De Hita, M.L. Costa

**Affiliations:** 1 Hospital Universitario José Germain, Hospital De Día, Leganes, Spain; 2 Hospital Universitario Jose Germain, Ucpp, Leganes, Spain; 3 Hospital Universitario Severo Ochoa, Psychiatry, Leganes, Spain

**Keywords:** hyperammonemic encephalopathy, valproate, side-effect

## Abstract

**Introduction:**

Hyperammonemic encephalopathy is an unusual side-effect of the treatment with valproic acid. According to several sources, this side effect could be underdiagnosed and underreported.

**Objectives:**

We present the case of a 54-year-old patient institutionalized in a psychiatric hospital who was referred to a general hospital after an episode of delirium of unknown etiology. The patient had been diagnosed with Schizoaffective disorder 26 years prior to his admittance and had started treatment with valproic acid the previous month.

**Methods:**

A case report is presented alongside a review of the relevant literature regarding valproate-induced hyperammonemic encephalopathy, its differential diagnosis and treatment.

**Results:**

During his hospital stay, the patient underwent a complete panel of tests including CT Scan, EEG, toxic panel and complete blood tests. In them the only altered parameter was hyperammonemia, therefore valproic acid was removed and was treated with lactulose and rifaximin to reduce ammonium levels.

**Conclusions:**

Hyperammonemic encephalopathy in the context of the treatment with valproic acid is a side-effect which is more prevalent than what was thought in the past. Valproate could reduce ammonium elimination therefore increasing the levels of the molecule. It should be administered carefully in cases where the patient may be presenting with other causes of increased ammonium metabolism or decreased elimination. It is important to bear in mind this possible side-effect to increase patient’s safety.

**Disclosure:**

No significant relationships.

